# Dual stimulation by autoantigen and CpG fosters the proliferation of exhausted rheumatoid factor-specific CD21^low^ B cells in hepatitis C virus-cured mixed cryoglobulinemia

**DOI:** 10.3389/fimmu.2023.1094871

**Published:** 2023-02-08

**Authors:** Martina Del Padre, Ramona Marrapodi, Ylenia A. Minafò, Eva Piano Mortari, Giovanna Radicchio, Chiara Bocci, Laura Gragnani, Alessandro Camponeschi, Stefania Colantuono, Lucia Stefanini, Stefania Basili, Rita Carsetti, Massimo Fiorilli, Milvia Casato, Marcella Visentini

**Affiliations:** ^1^ Department of Translational and Precision Medicine, Sapienza University of Rome, Rome, Italy; ^2^ Department of Molecular Medicine, Sapienza University of Rome, Rome, Italy; ^3^ B cell unit, Immunology Research Area, IRCCS Bambino Gesù Children’s Hospital, Rome, Italy; ^4^ Department of Experimental and Clinical Medicine, University of Florence, Florence, Italy; ^5^ Department of Rheumatology and Inflammation Research, Institute of Medicine, Sahlgrenska Academy, University of Gothenburg, Gothenburg, Sweden

**Keywords:** mixed cryoglobulinemia, rheumatoid factor, autoantigen, CD21^low^ B cells, exhaustion, BCR/TLR9 crosstalk

## Abstract

**Introduction:**

Hepatitis C virus (HCV) causes mixed cryoglobulinemia (MC) by driving clonal expansion of B cells expressing B cell receptors (BCRs), often encoded by the VH1-69 variable gene, endowed with both rheumatoid factor (RF) and anti-HCV specificity. These cells display an atypical CD21low phenotype and functional exhaustion evidenced by unresponsiveness to BCR and Toll-like receptor 9 (TLR9) stimuli. Although antiviral therapy is effective on MC vasculitis, pathogenic B cell clones persist long thereafter and can cause virus-independent disease relapses.

**Methods:**

Clonal B cells from patients with HCV-associated type 2 MC or healthy donors were stimulated with CpG or heath-aggregated IgG (as surrogate immune complexes) alone or in combination; proliferation and differentiation were then evaluated by flow cytometry. Phosphorylation of AKT and of the p65 NF-kB subunit were measured by flow cytometry. TLR9 was quantified by qPCR and by intracellular flow cytometry, and MyD88 isoforms were analyzed using RT-PCR.

**Discussion:**

We found that dual triggering with autoantigen and CpG restored the capacity of exhausted VH1-69pos B cells to proliferate. The signaling mechanism for this BCR/TLR9 crosstalk remains elusive, since TLR9 mRNA and protein as well as MyD88 mRNA were normally expressed and CpG-induced phosphorylation of p65 NF-kB was intact in MC clonal B cells, whereas BCR-induced p65 NF-kB phosphorylation was impaired and PI3K/Akt signaling was intact. Our findings indicate that autoantigen and CpG of microbial or cellular origin may unite to foster persistence of pathogenic RF B cells in HCV-cured MC patients. BCR/TLR9 crosstalk might represent a more general mechanism enhancing systemic autoimmunity by the rescue of exhausted autoreactive CD21low B cells.

## Introduction

Hepatitis C virus (HCV) causes monoclonal B cell lymphoproliferative disorders that include type 2 mixed cryoglobulinemia (MC) (1) and non-Hodgkin lymphomas (2). In both cases, monoclonal B cells express a restricted repertoire of stereotyped, or quasi-identical, B cell antigen receptors (BCRs) often encoded by the V_H_1-69/Vκ3-20 variable genes (3, 4). These stereotyped BCRs are endowed with both rheumatoid factor (RF) activity and specificity for HCV. The specificity for HCV is indicated by sequence homologies with human antibodies recognizing the E2 envelope protein of the virus (3, 4), although the reactivity of antibodies cloned from HCV-associated lymphomas with E2 or other HCV proteins has not been demonstrated so far (5). The strongest evidence for the dependence of these lymphoproliferative disorders on stimulation by HCV comes from the fact that the cure of infection with direct-acting antivirals (DAAs) leads in most patients to clinical improvement (6, 7). Nevertheless, cryoglobulins remain detectable in a substantial proportion of HCV-cured patients (6) and relapse of vasculitis occurs in about 12% of them (8), even many years after the clearance of virus and often in association with events characterized by increased production of immune complexes (ICs) such as infections or solid tumors (9).

The pathogenic B cells of MC patients display a peculiar phenotype characterized by low expression of CD21 (CD21^low^ B cells), by atypical expression of CD11c and of an array of homing and inhibitory surface receptors, and by impaired BCR signaling; also, proliferative responses to the triggering of BCR and of TLR9 are defective (10, 11). Phenotypically and functionally similar CD21^low^CD11c^pos^ B cells accumulate in HIV infection (12), in Sjögren’s syndrome (13), in a subset of common variable immunodeficiency (CVID) (14), and in aged mice where they are called age-associated B cells (15).

Intriguingly, large clones of pathogenic B cells persist in the blood of many MC patients long after the clearance of HCV infection by antiviral therapy (16), and probably underlie the HCV-independent relapses of vasculitis observed in a significant proportion of patients (8, 9). The pathogenic clonal B cells persisting after antiviral therapy progressively recover from some of their abnormal features, including BCR signaling defects, proneness to apoptosis and CD21^low^ phenotype, but remain impaired in their capacity to proliferate in response to TLR9 activation (17).

It is unclear how RF-specific pathogenic B cell clones can survive in MC patients in the absence of the HCV trigger, and it can be hypothesized that this may depend on stimulation by autoantigen. We previously observed that the simultaneous triggering of the BCR with anti-Ig antibody and of TLR9 with CpG resulted in proliferation of unresponsive clonal B cells of HCV-viremic MC patients (11). In the present work, we investigated whether autoantigen could mimic this effect of anti-Ig. Our results show that co-stimulation with CpG and autoantigen, in the form of surrogate IgG ICs, induces proliferation of RF-specific clonal B cells persisting in HCV-cured MC patients. This suggests that circulating ICs and microbial or cell-derived nucleic acids may cooperate to support the survival of pathogenic B cells in the absence of HCV.

## Methods

### Study subjects

For this study, we selected 13 patients with HCV-associated type 2 MC vasculitis who had circulating B cell clones identifiable by flow cytometry either through light chain restriction and distinctive immunophenotype or through the expression of V_H_1-69-encoded BCRs recognized by the G6 antibody, as previously described (11, 16, 17). All but one of the patients had been treated with DAA and had sustained virologic response, and all but one of virologic responders had clinical response of vasculitis as evaluated by previously reported criteria (6); one complete responder had subsequently a transient relapse of purpura. Six patients had B cell clones identifiable by the expression of V_H_1-69-encoded BCRs; further information on these patients is reported in **Supplementary Table 1**.

### Cells and immunophenotyping

Peripheral blood mononuclear cells (PBMCs) were obtained by density-gradient centrifugation; for some experiments B cells were purified by negative selection with immunomagnetic beads. Immunophenotyping was done using combinations of fluorochrome-labeled monoclonal antibodies (all from Becton-Dickinson Biosciences). The G6 monoclonal antibody, which recognizes an epitope of the V_H_1-69-encoded protein, was kindly provided by R. Jefferis, Birmingham, UK. Unlabeled G6 was counterstained with FITC- or PE-conjugated goat anti-mouse IgG (Becton-Dickinson Biosciences) using mouse IgG as control as previously described (11, 16, 17). Flow cytometric analyses were done on a FACSCalibur instrument (Becton-Dickinson Biosciences) using the CellQuest (Becton-Dickinson Biosciences) and FlowJo (Tree Star, Ashland, OR) software.

### Immune complexes and monomeric IgG

Surrogate ICs were generated, as described by Kobayashi et al. (18), by heath-aggregation of human monomeric IgG for 1 hour at 65°C followed by centrifugation at 10,000 x g for 2 min to remove precipitates. The source of monomeric IgG was a commercial preparation of intravenous immunoglobulin (IVIg) for therapeutic use (Kiovig^®^, Baxter, 100 mg/ml). Heat-aggregated and untreated IVIg were stored at 4°C and used for functional assays at a final concentration of 10 to 20 μg/mL.

### Proliferation and differentiation assays

Cell proliferation was measured by the carboxyfluorescein diacetate succinimidyl ester (CFSE) dilution assay. PBMC were labeled with CFSE (Invitrogen, Life Technologies), suspended in RPMI 1640 containing 10% fetal bovine serum and cultured at 2 × 10^5^ cells per well in 96-well U-bottom plates in the absence or presence of CpG ODN 2006 (2.5 μg/mL; *In vivo*gen), F(ab′)_2_ anti-human Ig (4 μg/mL; Jackson Immunoresearch Laboratories), ICs (heath-aggregated IVIg, 10 μg/mL) or monomeric IgG (IVIg, 10 μg/mL). At day 5 of culture cells were harvested and viability was assessed by small cell size and permeability to the non-vital dye 7-aminoactinomycin D (7-AAD); in several experiments fresh cells were co-stained with antibodies to CD20, IgM and V_H_1-69 (G6 antibody) (**Supplementary Figure 1**). Nonvital cells did not exceed 7% of lymphocytes without significant correlation with the stimulation conditions; we did not detect significant differences in the proportions of B cells or B cell subsets between viable and nonviable cells. After this preliminary analysis, cells were permeabilized (Permeabilizing-Solution-2; Becton-Dickinson Biosciences) to be able to investigate the cytoplasmic IgM content, and then stained with antibodies to CD20, IgM and V_H_1-69 and analyzed by flow cytometry. The proliferation parameters of electronically gated viable (region R2 in **Supplementary Figure 1**) CD20^pos^IgM^pos^ B cells, further gated into V_H_1-69^pos^ and V_H_1-69^neg^, were then evaluated by CFSE fluorescence dilution using the FlowJo software (Tree Star). The parameters taken into consideration were the “percent divided” (percentage of cells that started dividing calculated irrespective of the number of divisions), and the “proliferation index” (total number of divisions divided by the number of cells that went into division) which reflects the replicative capacity of the responding cells.

We also analyzed the differentiation of B cells induced by different stimuli by evaluating the frequency of cytoplasmic-IgM^high^CD20^low/neg^ cells; this population includes CD38^neg^ preplasmablasts, CD38^pos^ plasmablasts and CD138^pos^ plasma cells (19) and, therefore, we will collectively refer to these cells as IgM^high^CD20^low/neg^ differentiated B cells.

### BCR and TLR9 signaling assays

Phosphorylation of AKT and of the p65 NF-κB subunit was measured by flow cytometry using, respectively, the Phospho-Akt (Ser473) (D9E) XP^®^ Rabbit mAb #4060 labeled with Alexa Fluor 647 and rabbit IgG-Alexa Fluor 647 as control (Cell Signaling Technology), and the anti-NF-kB p65 phosphorylated at S529 [p65(pS529)] mouse mAb labeled with Alexa Fluor 488 (Becton-Dickinson), as per the manufacturer’s instructions. PBMC (1.5x10^6^ cells) were split in two vials, suspended in 100 μL of RPMI 1640 containing 5% fetal bovine serum (complete medium), and equilibrated at 37°C for 20 min. An equal volume of prewarmed complete medium, either alone (unstimulated control) or containing combinations of CpG (5 μg/mL), F(ab′)_2_ anti-human Ig (8 μg/mL), ICs (heath-aggregated IVIg, 20 μg/mL) or monomeric IgG (untreated IVIg, 20 μg/mL) was then added, and the cells were returned to 37°C for 20 min. Cells were then fixed by the addition of 200 μL of prewarmed Phos-Flow Fix Buffer I (Phos-Flow system, Becton-Dickinson Biosciences) for 10 min at 37^°^ C, washed twice in PhosFlow Perm/Wash Buffer I, split in two vials, and stained with the anti-pAkt or anti-p65(pS529) antibodies; samples were simultaneously stained with mAbs to CD19, IgM, CD27 or V_H_1-69 depending on experimental design. The pAkt-specific and the p65(pS529)-specific mean fluorescence intensity (MFI) was calculated by subtracting the MFI values obtained with fluochrome-conjugated rabbit or mouse control IgG from, respectively, those obtained with anti-pAkt or anti- p65(pS529) antibodies.

### Molecular characterization of BCRs

BCR clonality was assessed according to the “Biomed-2 concerted action for PCR-based clonality” guidelines as previously described (4). PCR products were run on non-denaturing 10% polyacrylamide gels and monoclonal bands were extracted and directly sequenced on an ABI PRISM 377-96 instrument (Perkin Elmer, Foster City, CA). The sequences obtained were submitted to the IMGT V-QUEST tool, and information on V-D-J/V-J genes and allele usage was extracted. The amino acid sequences deduced from the *IGHV*, *IGKV* and CDR3 DNA sequences were analyzed by the NCBI Basic Local Alignment Search Tool (BLAST) pairwise comparison program (https://blast.ncbi.nlm.nih.gov/Blast.cgi) for homologies with sequences of RFs and anti-HCV E2 antibodies from GenBank, as previously described (4); differences were evaluated by Fisher’s exact test (p ≤ 0.05 was considered significant).

### Quantification of TLR9 mRNA and protein and of MyD88 isoforms

TLR9 was quantified by qPCR and by intracellular flow cytometry. Intracellular content of TLR9 protein was analyzed by indirect staining of fixed/permeabilized cells with unlabeled anti-TLR9 mouse mAb (ab134368, Abcam), or control mouse IgG, counterstained with FITC-conjugated goat anti-mouse IgG; samples were simultaneously stained with mAbs to CD19, IgM, CD27 or other antibodies depending on experimental design; TRL9-specific fluorescence was calculated by subtracting the MFI values obtained with control mouse IgG from those obtained with anti-TLR9 antibody. For qPCR, purified (>95%) B cells were lysed with Trizol (Trizol^®^ Reagent, Applied Biosystem), and RNA was extracted according to the manufacturer’s instructions. Total RNA was retro-transcribed to cDNA using SuperScript™ VILO™ cDNA Synthesis Kit (Invitrogen). All samples were run in triplicate in a 15 μL reaction volume containing 2x TaqMan Universal PCR Master Mix (ThermoFisher Scientific), 20x primers from Integrated DNA Technologies (ACTB and TLR9), 30 ng of cDNA and water. The qPCR was run in the Abi Prism 7900 HT Fast Real Time PCR System (ThermoFisher Technologies) using the following amplification parameters: 10 min at 95°C followed by 40 cycles of 15 sec at 95°C, and 1 min at 60°C. Expression levels of TLR9 were calculated using the 2^-ΔΔCT^ method.

The relative amounts of differently spliced isoforms of MyD88, the canonical activating long isoform (MyD88_L_) and the short inhibitory isoform (MyD88_S_), were analyzed in semi-quantitative fashion using RT-PCR followed by agarose gel electrophoresis using the MyD88-L/S pair of primers described by De Arras and Alper (20): left TTGTTGGATGCCTGGCAGGGGCGCTCTGGC, right CACGGTCGGACACACACAACTTAAGCCGATAGTC.

### Statistics

Data were analyzed using the GraphPad Prism 9 software (La Jolla, CA, USA). Statistical differences were determined by using the Mann-Whitney U test for unpaired two groups, the paired t test, the Wilcoxon-Mann-Whitney test, or the nonparametric Friedman test; a significance level of p ≤ 0.05 was considered statistically significant.

## Results

### Co-stimulation by autoantigen and CpG drives the proliferation and differentiation of TLR9-unresponsive clonal B cells of MC patients

**Figure 1:**
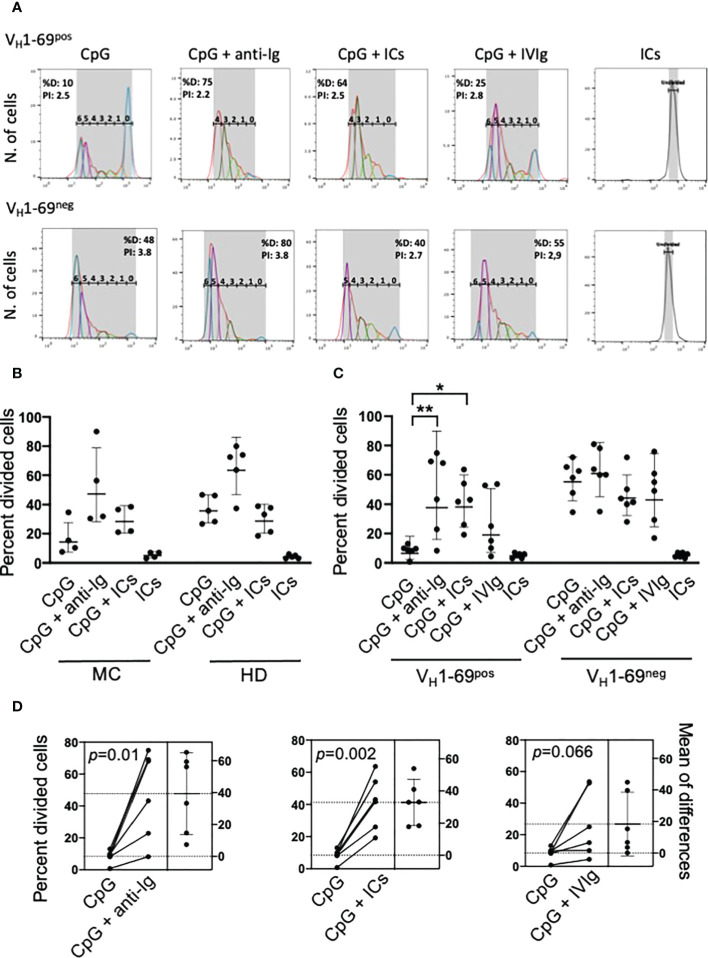
Co-stimulation with autoantigen (ICs) and CpG drives the proliferation of unresponsive RF-specific B cells from MC patients. **(A)** Representative analysis of the proliferation of electronically gated viable V_H_1-69^pos^ and V_H_1-69^neg^ B cells after different stimuli. The gated peaks of CFSE fluorescence define the number of cell divisions; %D (percent divided) is the number of cells that started dividing, and PI is the proliferation index. **(B)** Pilot experiments showing that stimulation with ICs and CpG, but not with ICs or CpG alone, induces the proliferation (expressed as percentage of cells that entered division, precursor cohort) of B cells from MC patients and not from healthy donors (HD). **(C)** ICs and not monomeric IgG (IVIg) increase CpG-induced proliferation of monoclonal V_H_1-69^pos^ and not of V_H_1-69^neg^ B cells of MC patients; *p=0.01, **p=0.001 by nonparametric Friedman test. **(D)** Estimation plots of changes in the percentage of divided V_H_1-69^pos^ B cells after different stimuli in individual patients; p-values are calculated by paired t test.

We previously reported that in HCV-viremic MC patients clonal V_H_1-69^pos^ B cells proliferated poorly in response to the stimulation of TLR9 with CpG or of the BCR with anti-Ig, while the simultaneous stimulation of TLR9 and BCR led a significant proportion of these cells to enter cell division (11). In this study, we investigated whether autoantigen, in the form of surrogate ICs generated by heath-aggregation of IgG, could reproduce this effect of anti-Ig on V_H_1-69^pos^ B cells identified by the G6 monoclonal antibody. The strategy for gating V_H_1-69^pos^ or V_H_1-69^neg^ IgM-expressing B cells is illustrated in **Supplementary Figure 1**, and the strategy of analysis of cell proliferation by the CFSE dilution method is illustrated in **Figure 1A**.

To confirm that the B cells identified by the G6 antibody, directed to the V_H_1-69 variable gene protein product, indeed expressed V_H_1-69-encoded heavy chains we characterized molecularly the BCRs of 3 of 6 patients. In all cases the expressed BCRs were encoded by V_H_1-69/V_k_3D-20 genes whose light chains carried complementarity determining region 3 (CDR3) sequences homologous to canonical RFs of MC patients as well as to antibodies against the E2 envelope protein of HCV (**Supplementary Table 2**), a signature of BCRs expressed in lymphoproliferative disorders secondary to HCV infection (3, 4).

We initially performed preliminary flow cytometry studies using whole B cells from HCV-cured MC patients and found that co-stimulation with ICs markedly increased CpG-induced proliferation of B cells from MC patients but not from healthy donors, whereas ICs alone were ineffective (**Figure 1B**). These experiments, however, did not allow to conclude that the proliferative response induced by ICs plus CpG was restricted to RF-producing B cells. Therefore, we performed further experiments in HCV-cured MC patients whose V_H_1-69^pos^ clonal B cells could be traced using the G6 antibody.

The frequencies of V_H_1-69^pos^ and V_H_1-69^neg^ IgM^pos^ B cells of MC patients that entered division after different stimuli are summarized in **Figure 1C**; in one patient these studies were repeated 10 months later with consistent results (not shown). The V_H_1-69^pos^ B cells proliferated poorly in response to CpG compared to autologous V_H_1-69^neg^ B cells (p=0.002), but co-stimulation with CpG and either anti-Ig or ICs significantly enhanced their proliferation; by contrast, co-stimulation with CpG and ICs had no effect on V_H_1-69^neg^ B cells. IVIg increased the proliferation of V_H_1-69^pos^ B cells in some patients, possibly because of the presence of some spontaneously aggregated IgG in the preparation used. **Figure 1D** displays the estimation plots of changes in the percentages of divided VH1-69pos B cells after different stimuli in individual patients. 

As the above results reflected only the frequency of B cells that entered division, we further analyzed the proliferation index (**Supplementary Figure 2**) that quite faithfully reflects the replicative capacity of dividing cells. V_H_1-69^pos^ B cells had a significantly reduced (p=0.007) proliferation index than V_H_1-69^neg^ cells upon stimulation with CpG, but co-stimulation with ICs significantly increased it (p=0.03). Co-stimulation with anti-Ig and, especially, with ICs resulted in proliferation indexes of V_H_1-69^pos^ B cells not significantly different of that of V_H_1-69^neg^ B cells co-stimulated with CpG and anti-Ig, suggesting that co-engagement of BCR and TLR9 may result in a near-normal replicative capacity of recruited V_H_1-69^pos^ B cells.

**Figure 2:**
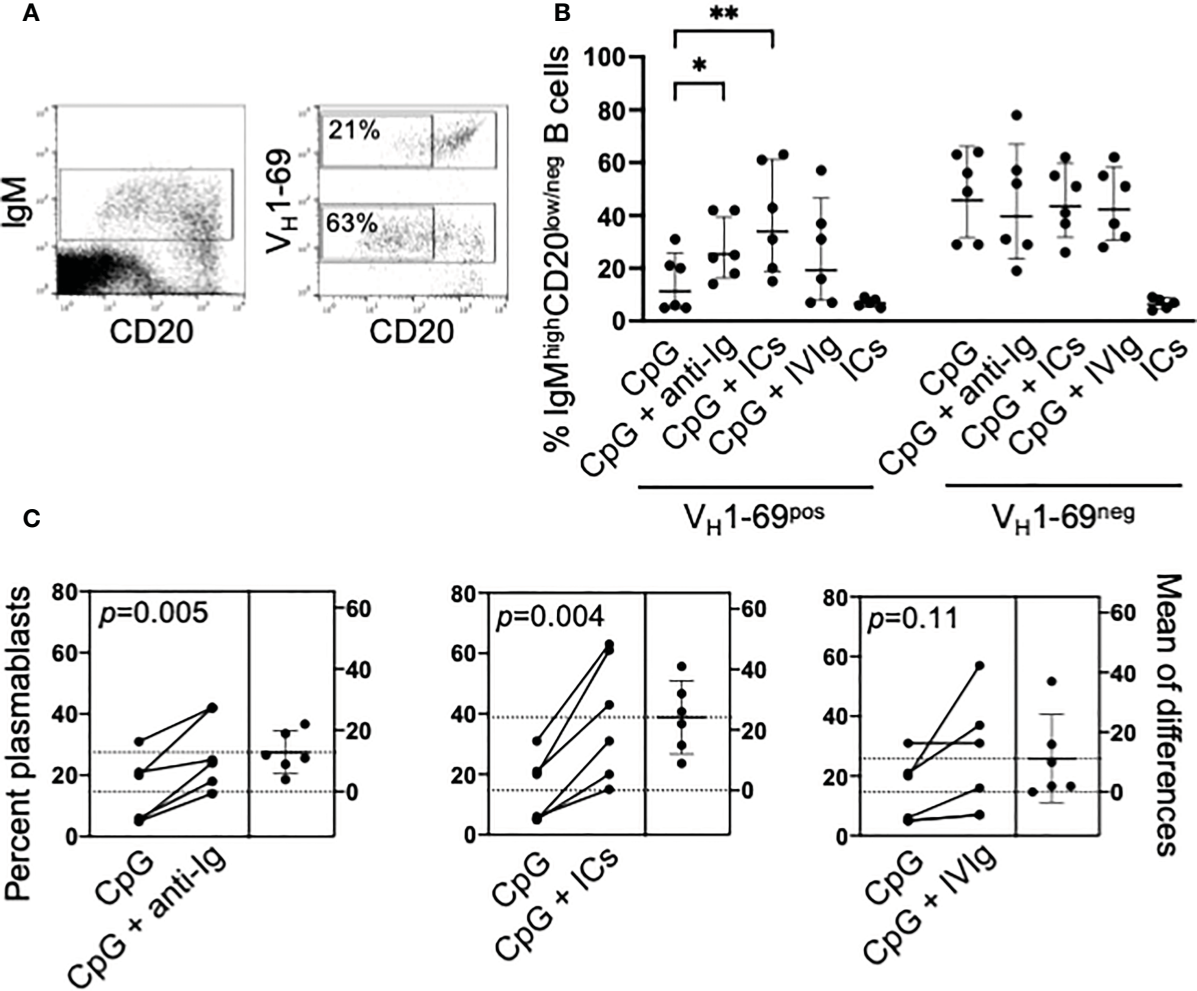
Co-stimulation with CpG and ICs drives the differentiation into IgM^high^CD20^low/neg^ B cells of unresponsive V_H_1-69^pos^ B cells. **(A)** Representative cytograms illustrating the gating strategy. PBMC from an MC patient were stimulated for 5 days with CpG and IgM^pos^ B cells were electronically gated; VH1-69^pos^ and VH1-69^neg^ cells were further gated and, in each population, the frequency of IgM^high^CD20^low/neg^ B cells was calculated. **(B)** Cumulative results of differentiation of VH1-69^pos^ and VH1-69^neg^ IgM B cells into IgM^high^CD20^low/neg^ B cells induced by different stimuli; *p=0.03, **p=0.0036 by nonparametric Friedman test. **(C)** Estimation plots of changes in the percentage of IgM^high^CD20^low/neg^ B cells after different stimuli in individual patients; p-values are calculated by paired t test.

We further investigated the differentiation of IgM^pos^CD20^pos^ B cells B cells into IgM^high^CD20^low/neg^ cells as illustrated in **Figure 2A**; it must be outlined that this strategy does not allow to discriminate preplasmablasts, plasmablasts and plasma cells (19). CpG-induced differentiation was significantly reduced (p=0.009) in V_H_1-69^pos^ compared to V_H_1-69^neg^ cells, and co-stimulation with anti-Ig or ICs significantly increased their differentiation whereas was ineffective on V_H_1-69^neg^ B cells (**Figure 2B**). As with proliferation, IVIg increased the differentiation of V_H_1-69^pos^ B cells in some patients (**Figure 2C**).

In summary, these data indicate that the co-stimulation of BCR by autoantigen and of TLR9 by CpG can promote the proliferation and the differentiation, the latter with the limitation of a lack of discrimination between different stages, of unresponsive RF-specific IgM^pos^ B cells of HCV-cured MC patients. To untangle the mechanism for this synergy we investigated the BCR and TLR9 signaling pathways in these cells.

### TLR9 and MyD88 are normally expressed in MC clonal B cells

**Figure 3:**
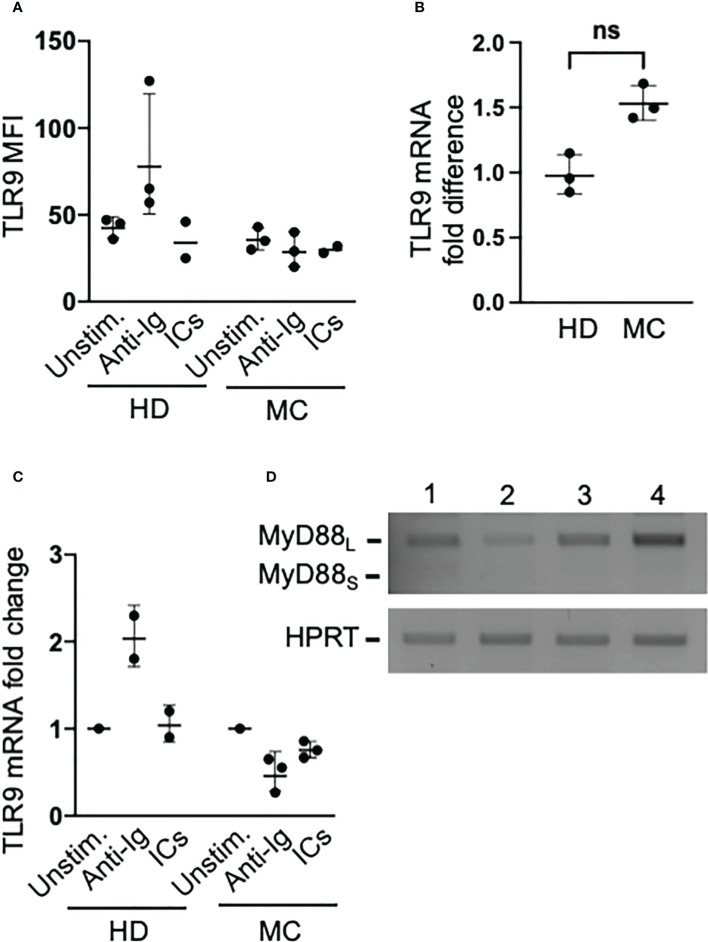
Clonal B cells of MC patients express abundant levels of TLR9 that are not increased by BCR signaling, and do not express the inhibitory MyD88_S_ isoform. **(A)** PBMC from MC patients with large (>80% of B cells) IgMκ^pos^CD27^pos^ clones and from healthy donors (HD) were cultured for 16 hours in the absence or presence of anti-Ig or ICs, and then stained with antibodies against CD20, IgM, CD27 and TLR9. TLR9 MFIs refer to IgM^pos^CD27^pos^ B cells in the two groups. **(B)** TLR9 mRNA levels measured by qPCR in freshly isolated purified whole B cells from MC patients with large B cell clones and from HD; ns, not significant p=0.1. **(C)** The B cells purified from MC patients and HD were cultured for 3 hours under the conditions indicated in panel A, and changes in mRNA were measured by qPCR. **(D)** The mRNAs from MC patients’ B cells used for the experiments depicted in panel B were used for semiquantitative RT PCR using primers that amplify both the agonist MyD88_L_ long isoform and the inhibitory MyD88_S_ short isoform of the MyD88 adaptor; results reveal a nearly exclusive usage of MyD88_L_ in these cells.

Since BCR triggering induces TLR9 expression in human naïve B cells (21), we asked whether induction of TLR9 expression by BCR signaling could explain the rescue of MC clonal B cells from unresponsiveness to CpG. In preliminary flow cytometry experiments using commercially available reagents, we concluded that best staining of TLR9 could be obtained with an unlabeled antibody that, however, prevented co-staining with unlabeled anti-V_H_1-69 antibody. Thus, we investigated TLR9 protein content in whole B cells from 3 HCV-cured MC patients with large (>80% of B cells) IgMκ^pos^ clones clearly identified by light chain restriction and CD21^low^ phenotype. We found that these patients’ clonal B cells constitutively expressed levels of TLR9 protein comparable to healthy donors’ B cells; however, unlike with normal B cells BCR triggering failed to increase their TLR9 protein content (**Figure 3A**). We next measured TLR9 mRNA content by qPCR in purified B cells from these three MC patients plus another patient also carrying a large B cell clone. TLR9 mRNA expression tended, although not significantly, to be higher in MC B cells than in those from healthy donors (**Figure 3B**) and, as observed with TLR9 protein, stimulation with anti-Ig increased TLR9 mRNA content only in healthy donors’ B cells (**Figure 3C**).

As upregulation of TLR9 did not appear to be involved in the capacity of BCR activation to induce proliferation of CpG-unresponsive MC B cells, we asked whether this phenomenon could be due to the rescue from a TLR tolerant state. Stimulation of a TLR renders immune cells unresponsive to subsequent TLRs stimulation, a process known as TLR tolerance, and this is particularly well studied in macrophages (21). Interestingly, human B cells stimulated *in vitro* with the TLR7/8 synthetic agonist R848 become unresponsive to the subsequent stimulation with R848 or with CpG but responsiveness to these ligands is restored by BCR signaling (22), thus closely recalling our findings.

A central mechanism of TLR tolerance in myeloid cells is the production of a short splice variant of MyD88 (MyD88_S_) that, unlike the agonist long form (MyD88_L_), antagonizes phosphorylation of IRAK and NF-κB activation (23). Since MyD88s is constitutively overexpressed by ex vivo TLR tolerant monocytes from patients with sepsis (24), it could be expected that pathogenic B cells of MC patients, if TLR tolerant, overexpress this splice variant. In contrast with this hypothesis, we found that only MyD88_L_ mRNA was expressed by these cells (**Figure 3D**). This finding, however, does not fully exclude that MC B cells are TLR tolerant, since TLR9 stimulation may not induce MyD88_S_ in human B cells (25) and other molecules can negatively regulate signaling pathways in TLR tolerance (21, 24, 26). Therefore, we investigated TLR9-induced activation of NF-κB, which is known to be inhibited in TLR tolerant B cells (22).

### Proficiency of TLR9-induced but not of BCR-induced signaling to NF-κB in MC B cells

**Figure 4:**
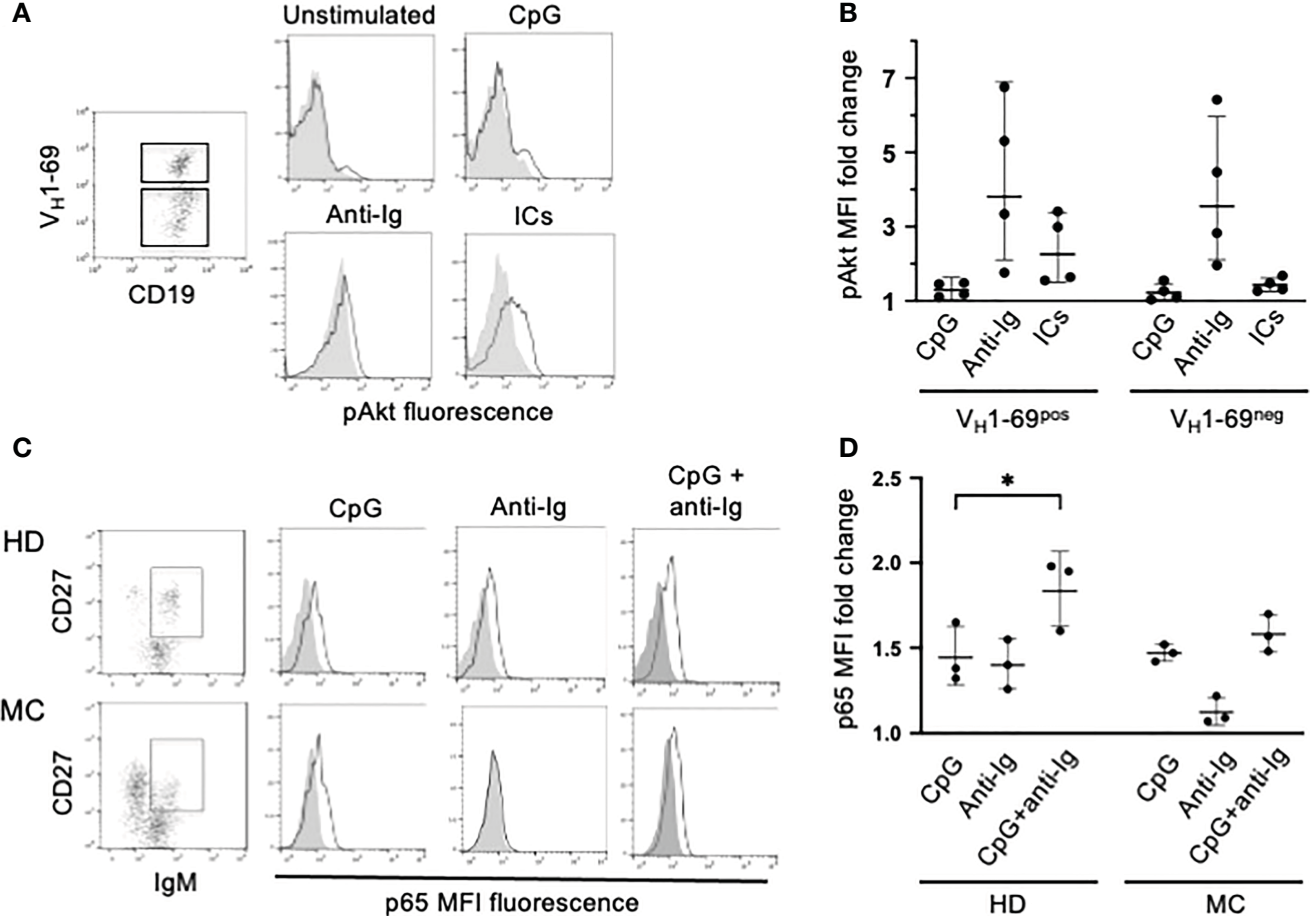
PI3K/Akt and NF-kB/p65 signaling in clonal B cells of MC patients. **(A)** PBMC from a representative MC patient were activated with different stimuli; electronically gated IgM^+^ B cells were further gated into V_H_1-69^pos^ and V_H_1-69^neg^ cell subpopulations and analyzed for pAkt-specific fluorescence (gray histograms denote V_H_1-69^neg^ and white histograms V_H_1-69^pos^ cells). **(B)** Cumulative results in 4 MC patients (results are expressed as MFI fold change compared to unstimulated cells) illustrating that ICs induce Akt phosphorylation selectively in V_H_1-69^pos^ cells. **(C)** PBMC from a representative MC patient with an IgMkCD27^pos^ B cell clone and from a healthy donor (HD) were left unstimulated or activated with different stimuli. After stimulation IgM^pos^CD27^pos^ cells were gated analyzed for changes in p65(pS529)-specific fluorescence (gray histograms denote unstimulated cells and white histograms stimulated cells). Results indicate that p65(S529) is phosphorylated after stimulation with CpG but not with anti-Ig in MC cells, while is efficiently phosphorylated after either stimuli in HD cells. **(D)** Cumulative results in 3 MC patients with circulating IgMkCD27^pos^ B cell clones and 3 in HDs, illustrating that IgM ^pos^CD27^pos^ clonal B cells of MC patients efficiently phosphorylate p65(S529) after stimulation with CpG but not with anti-Ig. *p=0.048 by paired t test.

We previously reported that BCR-driven phosphorylation of ERK is impaired in clonal B cells of HCV-viremic MC patients and normalizes shortly after the clearance of the virus by antiviral therapy (11, 17). Here we investigated BCR signaling through the PI3K/Akt pathway, which is required for BCR-induced rescue from TLR tolerance (22). We studied 4 MC patients and found that stimulation of the BCR with anti-Ig led to AKT phosphorylation both in V_H_1-69^neg^ and in V_H_1-69^pos^ B cells whereas ICs were effective, although less than anti-Ig, only on the latter cells (**Figures 4A, B**). These data suggest that the PI3K/Akt pathway is intact in RF-specific B cells of HCV-cured MC patients and that can be activated by autoantigen; thus, in principle autoantigen could be able to induce rescue from TLR tolerance through BCR signaling. To try to untangle this issue we investigated TLR signaling in MC clonal B cells.

TLR7/TLR9 tolerance in human B cells is associated with reduced activation of the canonical NF-κB signaling pathway by these TLRs (22). Thus, we investigated TLR9- and BCR-induced phosphorylation of the p65 NF-κB subunit at serine 529 [p65(pS529)]. We choose this signature of p65 activation because it hallmarks synergy between TLR9 signaling, cytokine milieu and BCR signaling in promoting clonal expansion of chronic lymphocytic leukemia cells (27, 28), and because the BCR-mediated phosphorylation of p65(pS529) is defective in CD21^low^ B cells of CVID patients (29). For these experiments we investigated whole B cells from MC patients harboring B cell clones that did not express V_H_1-69 but were readily identifiable by a distinctive IgMκCD27^pos^ mostly CD21^low^ phenotype (not shown). Stimulation with CpG resulted in a similar increase of p65(pS529) phosphorylation in CD27^pos^ B cells from healthy donors and MC patients whereas p65(pS529) phosphorylation induced by anti-Ig was defective in MC clonal B cells; co-stimulation with CpG and anti-Ig tended to have an additive effect on p65(S529) phosphorylation in healthy donors’ B cells but not in MC B cells (**Figures 4C, D**).

In summary, TLR9-induced, and not BCR-induced, activation of NF-kB is proficient in MC B cells, strongly arguing against a state of TLR tolerance (22).

## Discussion

The IgM antibodies expressed by MZ B cells clonally expanded in HCV-associated MC are polyreactive and are reputedly endowed both with RF activity and with specificity for a HCV antigen, possibly the E2 envelope protein (1–5). The strongest argument in favor of the dependence of the proliferation of pathogenic B cell clones of MC patients on continual antigenic stimulation by HCV is that, in most cases, vasculitis regresses upon the eradication of infection by antiviral therapy (6, 7). As an alternative to direct antigenic stimulation by the virus, it has been suggested that HCV-bound IgG might be the major driver of proliferation of these RF-producing B cells (1, 30), but this model hardly explains why in other chronic infections, for example in HIV infection where there is also an accumulation of virus-specific CD21^low^CD11c^pos^ B cells (12), there is no expansion of RF-expressing B cell clones.

The RF-specific clones found in MC patients express a restricted set of stereotyped BCRs, which are different from those encoding RFs in rheumatoid arthritis (31) but are highly homologous to those expressed by CD21^low^CD11c^pos^ B cells clonally expanded in primary Sjögren’s syndrome, another lymphoproliferative disorder of RF-specific B cells (13, 32, 33). It has been suggested that in Sjögren’s syndrome ICs formed by anti-Ro/SSA and anti-La/SSB autoantibodies drive the clonal expansion of RF B cells (34). Furthermore, the BCRs of clonal B cells of MC patients are also highly homologous to those expressed by neoplastic B cells in a subset of RF-producing chronic lymphocytic leukemia putatively dependent on autoantigen (4, 35–38). Thus, few RF-specific stereotyped BCRs seem to be uniquely associated with vigorous cellular response to autoantigen, namely IgG ICs, leading to diverse lymphoproliferative disorders.

We found that the TLR9-driven proliferation and differentiation of clonal RF-specific B cells of MC patients was defective compared to allogeneic or autologous normal B cells. At variance with our findings, Comarmond and colleagues (39) recently reported that TLR9 stimulation with CpG induced the secretion of proinflammatory cytokines as well as the proliferation of CD21^low^ B cells from patients with HCV-associated MC. The latter statement, however, was supported by only one representative experiment without comparison with normal CD21^pos^ B cells; furthermore, the same study showed that CpG induced extensive apoptosis of CD21^low^ B cells, contrasting with well-preserved proliferative responses. A previous study reported (13) that RF-producing CD21^low^ B cells of patients with Sjögren’s syndrome, which are phenotypically and immunogenetically (32, 33) very similar to those found in MC, proliferated poorly in response to CpG when compared to their CD21^pos^ normal counterparts. Furthermore, the CD21^low^ B cells expanded in HIV infection or in CVID, which phenotypically and functionally closely resemble those of MC patients, proliferate poorly in response to CpG (12, 14). Remarkably, co-stimulation of BCR and of TLR9 in the presence of T cell help largely restored the proliferation of CD21^low^ B cells from HIV-infected patients (12), indicating that also in this disease inhibitory pressure can be overridden.

**Figure 5:**
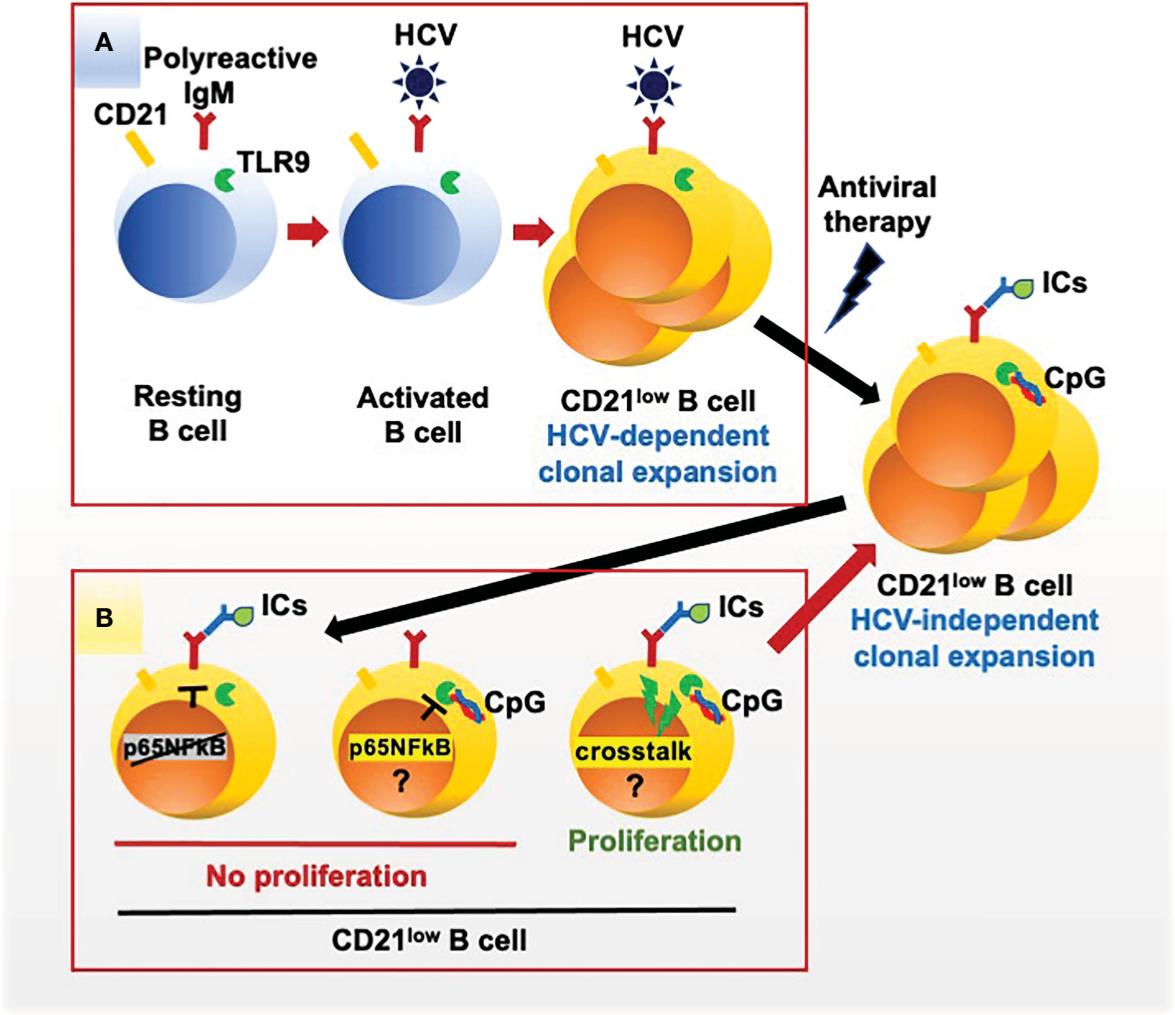
HCV-dependent and autoantigen-dependent expansion of rheumatoid factor B cell clones in mixed cryoglobulinemia. **(A)** HCV drives the proliferation of clones of B cells expressing a polyreactive BCR endowed with anti-HCV and rheumatoid factor specificities; the expanded B cell clones acquire a peculiar CD21^low^ phenotype and functional exhaustion. **(B)** Once HCV is cleared by antiviral therapy CD21^low^ B cell clones survive for a long time; these exhausted cells fail to proliferate in response to the engagement of the BCR by autoantigen (immune complexes, ICs) and of TLR9 by CpG but proliferate in response to the simultaneous engagement of both receptors, possibly supporting the virus-independent survival of pathogenic CD21^low^ B cell clones *in vivo*. Phosphorylation of the NF-kB p655 subunit is deficient after BCR stimulation and proficient after TLR9 stimulation; thus, the mechanisms for reduced proliferation of CD21^low^ B cell clones after TLR9 stimulation and for the BCR/TLR9 positive crosstalk remain undefined.

Our findings provide evidence that ICs and CpG may concur to induce the proliferation of unresponsive RF-producing clonal B cells from HCV-cured MC patients (**Figure 5**). On a clinical ground, this may explain why HCV-independent relapses of MC vasculitis occur often in concomitance with events, such as infections or cancer, which determine abundant production of ICs and of stimulatory DNA of microbial or cellular origin (9). Thus, in a healthy condition continual low-level stimulation by physiologic levels of ICs and of microbial or apoptosis-related nucleic acids may support survival of pathogenic B cell clones (16), whereas overproduction of these ligands may over-activate cells leading to clinical relapse. Low-level stimulation of BCR and TLR9 would seemingly provide proliferative signals weaker than and qualitatively different from those provided by HCV through the simultaneous activation of BCR by a viral antigen, of TLR7 by single stranded RNA and of the CD81 coreceptor by the E2 envelope protein (40); this may explain why MC clonal B cells surviving after antiviral therapy progressively loose their CD21^low^ phenotype and recover from some functional defects although remain unresponsive to CpG (17).

We tried to untangle the BCR/TLR9 signals that override unresponsiveness of MC B cells but failed to depict a consistent scenario. Our findings may argue against a rescue from TLR tolerance (22) since, in the face of a proliferative defect, the TLR9/NF-κB signaling pathway appeared intact based on the phosphorylation of p65(S529). However, anomalies in p65 phosphorylation at sites other than S529 or in the phosphorylation of other NF-κB subunits might be responsible for defective signaling (41).

Impaired BCR-mediated NF-κB signaling through the canonical pathway (p65(S529) phosphorylation and IκBα degradation), with relatively preserved TLR9-mediated NF-κB signaling, was described in the CD21^low^ B cells of patients with CVID or with HIV infection (29). Thus, defective BCR signaling to NF-κB was suggested to represent a common feature of CD21^low^ B cells irrespective of the underlying disorder, and our findings support this view.

An elegant study on class switch recombination (CSR) in B cells (42) showed that isolated signaling by TLR9 (or other TLRs) and by the BCR resulted in marginal or no CSR, whereas combined signaling resulted in robust CSR; thus, it was concluded the BCR and TLRs may complement each other and enhance reciprocally their pathways of activation by engaging the canonical and non-canonical NF-κB pathways.

Other signaling pathways could be involved in the synergistic BCR/TLR9 crosstalk in MC B cells. The adaptor dedicator of cytokinesis-8 (DOCK8) is phosphorylated upon TLR9 activation, and then binds the Lck/Yes novel tyrosine kinase (Lyn) leading in turn to phosphorylation of the spleen tyrosine kinase (Syk), activation of STAT3 and B cell proliferation (43). Interestingly, the CD21^low^ B cells of patients with CVID or HIV infection, which closely resemble those of MC patients, constitutively overexpress Syk but have reduced activation of Syk and of downstream signaling molecules upon BCR stimulation (44). Thus, the DOCK8-dependent phosphorylation of Syk, independent from that induced by BCR, might contribute to restore the signaling cascade. Another molecule important for synergy between BCR and TLR9 is transforming growth factor beta activated kinase 1 (TAK1). TLR9 signaling leads to activation of IRAK1 that, in collaboration with Syk, recruits TNF receptor-associated factor 6 (TRAF6) to activate TAK1 (45), which plays an essential function in innate and adaptative responses by activating NF-kB and MAPKs (46) and is central in orchestrating synergy between TLR9 and BCR for proliferation, differentiation and antibody production (47). Therefore, DOCK8 and TRAF6 could combine to boost BCR-induced Syk and TAK1 activation in MC B cells.

## Conclusions

We demonstrate that the simultaneous engagement of BCR by autoantigen in the form of surrogate ICs and of TLR9 by CpG drives the proliferation and differentiation of otherwise unresponsive RF-specific pathogenic B cells from patients with HCV-cured of MC. This provides an explanation for the long-lasting persistence in many HCV-cured MC patients of pathogenic B cell clones that can cause virus-independent relapses of vasculitis.

We acknowledge that our study has limitations. An important one is the small number of patients in some experiments; this was because of the rarity of MC patients with the characteristic of clearly identifiable clones of pathogenic B cells long after the cure of HCV infection. Another limitation is the lack of identification of different stages of differentiation from preplasmablasts to plasma cells and of class switch induced in clonal B cells by different stimuli.

It is of interest that relapses of MC vasculitis are often associated with events, such as bacterial infections or cancer, that cause an increase of circulating ICs and of TLR9 ligands (9). Seminal studies by Marshak-Rothsteins’ group showed that dual engagement of BCR and TLR7/TLR9 is critical for the initiation of systemic autoimmunity (48, 49). Our results point to an important role for BCR/TLR9 crosstalk in the perpetuation of autoimmunity by counteracting unresponsiveness of each of these receptors that occurs when activated autoreactive B cells evolve into functionally exhausted CD21^low^ B cells.

## Data availability statement

The original contributions presented in the study are included in the article/**Supplementary Material**. Further inquiries can be directed to the corresponding author.

## Ethics statement

The studies involving human participants were reviewed and approved by Ethics Committee of Sapienza University of Rome Prot.n. 678/18. The patients/participants provided their written informed consent to participate in this study.

## Author contributions

MV, MF, and MC designed the study. MDP, RM, YAM, EP, GR, and CB performed the laboratory studies. MV, MF, RC, and LS supervised methodology, data curation and statistical analysis. LG, AC, SC, and LS contributed to the study development. MV and MF drafted the manuscript, figures and tables. LG, AC, SC, LS, SB, RC, and MC critically reviewed the manuscript and contributed to the conclusions. All authors contributed to the article and approved the submitted version.
