# Erratum: Dual stimulation by autoantigen and CpG fosters the proliferation of exhausted rheumatoid factor-specific CD21^low^ B cells in hepatitis C virus-cured mixed cryoglobulinemia

**DOI:** 10.3389/fimmu.2024.1369683

**Published:** 2024-01-26

**Authors:** Frontiers Production Office

**Affiliations:** Frontiers Media SA, Lausanne, Switzerland

**Keywords:** mixed cryoglobulinemia, rheumatoid factor, autoantigen, CD21^low^ B cells, exhaustion, BCR/TLR9 crosstalk

Due to a production error, the originally published document included a mistake in the affiliation 3. Instead of “B cell unit, Immunology Research Area, IRCCS Bambino Gesù Children’s Hospital, Florence, Italy”, the correct affiliation is “B cell unit, Immunology Research Area, IRCCS Bambino Gesù Children’s Hospital, Rome, Italy”.

The publisher apologizes for this mistake.

The original version of this article has been updated.

