# Supplementary material for: Dual stimulation by autoantigen and CpG fosters the proliferation of exhausted rheumatoid factor-specific CD21low B cells in hepatitis C virus-cured mixed cryoglobulinemia
Source: Front Immunol. 2023 Feb 8;14:1094871. doi: 10.3389/fimmu.2023.1094871 (PMC9945227; doi:10.3389/fimmu.2023.1094871)
Supplement: Supplementary file 1 [file DataSheet_1.docx]

**Supplementary material**

**Supplementary Table I**. Demographic and clinical data of patients with circulating V_H_1-69^pos^ B cell clones. The response of vasculitis was evaluated as previously described (6).

| **Pt** | **Age/**  **sex** | **Time of the study (months after DAA)** | **Percent among total B cells and absolute number of circulating V_H_1-69^pos^ B cells** | | **Cryocrit (percent)** | | **Symptoms before DAA*** | **Clinical response of vasculitis*** |
| --- | --- | --- | --- | --- | --- | --- | --- | --- |
|  |  |  | ***Before DAA*** | ***Time of***  ***study*** | ***Before DAA*** | ***Time of study*** |  |  |
| **1** | 61/F | 30 | 88% (153/μL) | 46% (57/μL) | 3 | 0 | P, N, A | CR** |
| **2** | 79/F | 29 | 81% (363/μL) | 52% (120/μL) | 2 | 1 | P, N, KD | CR |
| **3** | 81/F | 10 | 15% (170/μL) | 38% (344/μL) | 9 | 0 | P, N, A | CR |
| **4** | 86/M | untreated |  | 37% (58/μL) | 2 | N/A | P | N/A |
| **5** | 76/F | 10 | 15% (45/μL) | 25% (61/μL) | 4 | 0 | N, A | CR |
| **6** | 64/F | 24 | 95% (211/μL) | 93% (115/μL) | 11 | 5 | P, N, A | NR |

** A, arthralgia; KD, kidney disease; N, neuropathy; P, purpura. CR, complete response; NR, no response; N/A, not applicable.*

*** The study was done during complete response, which was followed by transient relapse of purpura.*

**Supplementary Table 2. (A)** Heavy (HCDR3) and kappa light chain (KCDR3) complementarity determining region (CDR3) sequences of clonal B cells from 3 MC patients. (**B**) Sequence homologies of patients’ HCDR3s and KCDR3s with RFs and anti-HCV E2 protein antibodies.

**A**

| **Pt** | **IGHV gene** | **HCDR3** | **GenBank**  **Accession #** |
| --- | --- | --- | --- |
| 1 | VH1-69 | AREGRGTVTTNPFDY | MN974489 |
| 2 | VH1-69 | ARGFSPLGDSSGYYYAY | MN974490 |
| 3 | VH1-69 | AREGRSGYVNPFDY | MN974491 |
|  | **IGKV gene** | **KCDR3** |  |
| 1 | VK3D-20 | QQYGSSPQT | MN974502 |
| 2 | VK3D-20 | QQYGSSPYT | MN974503 |
| 3 | VK3D-20 | QQYGSSPGT | MN974504 |

| **Pt** | **RF homology*,**  **E-value and**  **% of identity** | **Anti-HCVE2 homology*,**  **E-value and**  **% of identity** |
| --- | --- | --- |
|  | **HCDR3** | |
| 1 | RF-BOR, 5e^-07^ (67%) | No |
| 2 | RF-WOL, 3e^-03^ (47%) | No |
| 3 | RF-BOR, 1e^-07^ (71%) | No |
|  | **KCDR3** | |
| 1 | RF-CUR, 3e^-07^ (89%) | 3e^-07^ (89%) |
| 2 | RF-FLO, 5e^-09^ (100%) | 7e^-08^ (89%) |
| 3 | RF-CUR, 2e^-06^ (100%) | 2e^-06^ (100%) |

**B**

** The following sequences (GenBank accession numbers) were used for homology comparisons. Rheumatoid factors. HCDR3: RF-BOR 1313976A, RF-WOL 0707281c; KCDR3: RF-CUR 1206991°, RF-FLO 1206991B. Anti-HCV E2 antibodies. KCDR3: CAB43012.1, CAB43013.1.*

**Supplementary Fig. 1**. Strategy for the electronic gating of IgM^pos^/V_H_1-69^pos^ and IgM^pos^/V_H_1-69^neg^ B cells. PBMC from a representative MC patient were stimulated for 5 days with CpG and then stained with fluochrome-conjugated anti-CD20 and anti-IgM antibodies, with unlabeled anti-V_H_1-69 G6 antibody (indirect staining with fluochrome-conjugated goat-anti mouse antibody after blocking with normal mouse serum), and with the non-vital dye 7-aminoactinomycin D (7-AAD). Nonviable cells (small cells mostly 7-AAD^pos^) are gated in R1 (6%), whereas viable (>99% 7-AAD^neg^) lymphocytes, including large blast-like cells, are gated in R2 (64%). The IgM^pos^ B cells contained in R2 are gated and further subdivided into V_H_1-69^pos^ and V_H_1-69^neg^ cells; in these subpopulations, the relative proportions of CD20^bright^ B cells and of CD20^dim/neg^ plasmablasts can be calculated (see Fig.2A in the main manuscript).

**Supplementary Fig. 2**. Proliferation indexes of V_H_1-69^pos^ and V_H_1-69^neg^ IgM-expressing B cells from 6 patients with MC after different stimuli. Statistical differences are calculated by paired (within cell type) or unpaired (between cell types) t-test.
